# Posterosuperior and Anterior Rotator Cuff Repairs Yield Comparable 1‐Year Constant Scores in Routine Clinical Practice: A Propensity Score‐Matched Study From LaTour Group

**DOI:** 10.1002/ars2.70104

**Published:** 2026-10-09

**Authors:** Mustafa S. Rashid, Hugo Bothorel, Alberto Guizzi, Philippe Collin, Arash Amiri, Jeanni Zbinden, Alexandre Lädermann

**Affiliations:** ^1^ East Suffolk and North Essex NHS Foundation Trust Colchester Essex UK; ^2^ Research Department La Tour Hospital Meyrin Switzerland; ^3^ Department of Medical and Surgical Specialties, Radiological Sciences, and Public Health University of Brescia Brescia Italy; ^4^ CHP Saint‐Gregoire Saint‐Grégoire France; ^5^ Clinique Victor Hugo Paris France; ^6^ American Hospital of Paris Neuilly‐sur‐Seine France; ^7^ Trauma Research Center, Rajaee (Emtiaz) Trauma Hospital Shiraz University of Medical Sciences Shiraz Iran; ^8^ Division of Orthopaedics and Trauma Surgery Hôpital de La Tour Meyrin Switzerland; ^9^ Division of Orthopaedics and Trauma Surgery, Department of Surgery Geneva University Hospitals Geneva Switzerland; ^10^ Faculty of Medicine University of Geneva Geneva Switzerland; ^11^ FORE (Foundation for Research and Teaching in Orthopedics, Sports Medicine, Trauma, and Imaging in the Musculoskeletal System) Meyrin Switzerland

## Abstract

**Purpose:**

To compare clinical outcomes and ultrasound‐assessed healing rates between posterosuperior and anterior rotator cuff repairs using propensity score matching.

**Methods:**

Consecutive patients undergoing rotator cuff repair between 2015 and 2021 by a single fellowship‐trained shoulder surgeon were included if they had posterosuperior or anterior rotator cuff tears, Goutallier fatty infiltration grade 0 to 2, and a follow‐up of 1 year. Revision surgery, anterosuperior tears, and partial repairs were exclusion criteria. Propensity score matching (1:3) was performed on age, sex, traumatic origin, tendon retraction, and preoperative internal rotation and Constant scores. The primary outcome was the 1‐year Constant score. Secondary outcomes included range of motion, visual analog scale, American Shoulder and Elbow Surgeons, Single Assessment Numeric Evaluation, and ultrasound‐assessed healing (Sugaya classification) at 6 months.

**Results:**

Of the 1045 screened patients, 639 met the inclusion criteria. After propensity score matching, 53 patients with anterior cuff tears were matched to 146 patients with posterosuperior tears, with excellent covariate balance between groups. Postoperative patient‐reported outcome measures (Constant, American Shoulder and Elbow Surgeons, Single Assessment Numeric Evaluation, and pain) and range of motion at 1 year were similar between groups, and ultrasound‐assessed healing at 6 months did not differ. The proportions of patients achieving or exceeding the minimal clinically important difference were also comparable between groups for all outcome measures, including the Constant score (76% vs 81%), American Shoulder and Elbow Surgeons score (69% vs 66%), pain visual analog scale (92% vs 96%), and Single Assessment Numeric Evaluation score (77% vs 85%) (*P* = .569, *P* = .823, *P* = .525, and *P* = .291, respectively). In the adjusted mixed‐effects model, tear location was not statistically associated with the postoperative Constant score (*β* = 1.7, 95% confidence interval −3.0 to 6.3; *P* = .485).

**Conclusions:**

In this propensity score‐matched cohort, anterior and posterosuperior arthroscopic rotator cuff repairs in patients with limited fatty infiltration yielded comparable 1‐year Constant scores in a real‐world setting.

**Level of Evidence:**

Level III, retrospective comparative study.

## Graphical abstract

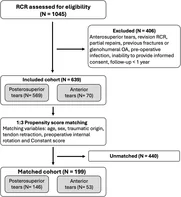

Posterosuperior cuff tears have been well investigated in the scientific literature, on account of being frequently encountered. Subscapularis tear involvement is less commonly seen, accounting for around 30% of primary rotator cuff repairs (RCRs) and 10% in isolation.^1^ There is debate within the shoulder surgery community regarding differences in clinical outcomes and healing rates of various rotator cuff tear patterns. Some believe that anterior RCRs heal more often than posterosuperior cuff tears. However, few studies have investigated this hypothesis. Although some factors have been well studied, such as tear size,^2^ fatty infiltration,^3^ tendon retraction,^4^ and age,^5^ there is little evidence evaluating the differences in tear location on clinical and radiological outcomes.^6^

Posterosuperior rotator cuff lesions are more common^7^ and tend to occur in older patients suffering from degenerative conditions.^8^ Anterior tears involving the subscapularis tendon are thought to occur in higher prevalence in younger patients, with greater likelihood of traumatic origins,^8^ better vascularization,^9^ and less tendon retraction.^10^ Approximately 14% of the total strength of the rotator cuff is generated by the supraspinatus,^11^ as opposed to the 53% provided by the subscapularis tendon. Given these differences, anterior RCR may better restore anterior force balance and yield superior structural and functional outcomes compared with posterosuperior RCR.^6^

The purpose of this study was to compare clinical outcomes and ultrasound‐assessed healing rates between posterosuperior and anterior RCRs using propensity score matching. The hypothesis was that anterior RCRs would have better clinical, functional, and structural outcomes than posterosuperior RCRs.

## METHODS

### Study Design and Patient Selection

All patients undergoing RCR between January 2015 and September 2021, performed by the senior author (A.L.), were assessed for eligibility for inclusion in this retrospective cohort study. Data were extracted from a prospective RCR database (Follow Health, Rennes, France), the use of which had been ethically approved (Association des médecins du canton de Genève et société médicale #12‐26). The inclusion criteria included patients aged 18 to 80 years, posterosuperior tears or anterior (isolated subscapularis) full‐thickness RCR, and rotator cuff muscle fatty infiltration limited to Goutallier grade 0 to 2.^12^ Patients with the following conditions were excluded: anterosuperior rotator cuff tears, revision RCR, shoulder pathologies such as previous fractures or progressive glenohumeral osteoarthritis, preoperative infection, inability to provide informed consent, partial repairs, and follow‐up time of less than 1 year. The eligible patients were divided into 2 groups based on tear location (posterosuperior vs anterior cuff tears).

### Institutional Review Board

This study was approved by the local ethics committee (CCER 2015‐15 trial registration number NCT02725346, CCER 2016‐00818 trial registration number NCT02943005, and 2019‐02076 trial registration number NCT04321005),^13^ and patients provided written informed consent for their participation and use of their data and images for research purposes.

### Study Variables

The primary outcome was the Constant score evaluated at 1 year postoperatively.^14^ Secondary outcomes included rotator cuff tendon healing determined by ultrasound^15^ at the 6‐month^16^, ^17^ postoperative follow‐up, as well as pain and functional scores (American Shoulder and Elbow Surgeons [ASES]^18^ and Single Assessment Numeric Evaluation [SANE]^19^) at 1‐year follow‐up. The following baseline characteristics were assessed: age, sex, side, laterality, medical comorbidities (such as diabetes mellitus, hypertension, and hypercholesterolemia), tobacco use, delay between symptom onset and surgical treatment, Workers’ Compensation status, shoulder range of motion, cause (traumatic vs atraumatic), tear location, and retraction level of tears.

### Surgical Technique for Posterosuperior and Anterior RCRs

All patients underwent surgery by the senior author (A.L.). Patients were positioned in the beach‐chair position, with an interscalene nerve block as well as general anesthesia. A standard posterior viewing portal was used for glenohumeral joint inspection, and the biceps tendon was addressed according to the presence of biceps pathology. Tenodesis or tenotomy, where indicated, was left to the discretion of the surgeon.^20^, ^21^ The rotator cuff tear was identified, debrided, and released if needed. The number of anchors and surgical technique repair were determined by the size of the tear present. The posterosuperior rotator cuff tears were repaired using either a single‐row repair or an independent double‐row technique.^22^ The surgical technique employed for the anterior lesions consisted of a single‐row repair with mattress sutures inferiorly and a lasso‐loop superiorly. Adjunct acromioplasty was performed in patients who had radiographic signs of dynamic impingement or to improve visualization during the procedure.^23^, ^24^, ^25^ Resection of the distal part of the clavicle was performed on patients who presented preoperatively painful acromioclavicular joints on palpation.

### Postoperative Rehabilitation

All patients received an “à la carte” postoperative rehabilitation protocol.^26^ When the quality of the tendon was suboptimal, immobilization by sling was proposed for 4 to 6 weeks.^27^ A sling was systematically proposed for anterior repairs.^28^ No sling was proposed for patients who undergo posterosuperior repair without tension.^29^, ^30^ Patients were allowed mobilization as tolerated with the elbow at the side. A range‐of‐motion program was then commenced at 6 weeks postoperatively, either aquatic therapy or standard rehabilitation, for all types of postoperative protocols.^31^

### Clinical and Radiological Outcome Measurements

At the preoperative and postoperative (6 months and 1 year) examinations, the range of motion, pain visual analog scale, Constant score,^14^ SANE,^19^ and ASES^18^ shoulder scores were evaluated. Range of motion and strength measurements were conducted by independent assessors who were not part of the current study, under the supervision of the senior author (A.L.), ensuring the reproducibility of the methodology used. Anterior forward flexion, external rotation and internal rotation (IR) were measured. External rotation was measured with the arm by the side of the body, and IR was assessed at the highest vertebral spinous process attained by the extended thumb of the patient. The level of the vertebral spinous process was assessed via manual counting from the C7 vertebra. The other scores were selected for their ease of administration in addition to their validated performance of shoulder function.^32^

Intraoperative rotator cuff tear pattern was classified according to the Collin classification.^6^ The latter divides the rotator cuff into 5 components: supraspinatus, superior subscapularis, inferior subscapularis, infraspinatus, and teres minor. Among them, type D represents supraspinatus and infraspinatus lesions, and type E represents supraspinatus, infraspinatus, and teres minor involvements. The present study compared isolated subscapularis lesions with isolated supraspinatus, D‐ and E‐type lesions. Preoperative imaging by magnetic resonance imaging was also assessed for fatty infiltration, staged according to the Goutallier classification^12^ and rotator cuff retraction, graded by the Patte classification.^33^ Healing of the repaired cuff was evaluated by ultrasound according to the modified Sugaya classification.^34^ All ultrasounds were performed at 6 months postsurgery^16^, ^17^ by the senior surgeon (A.L.) following a published systematic protocol.^15^ Ultrasound was considered the imaging modality of choice for its accessibility, cost, and accuracy for determining postoperative rotator cuff healing.^35^ Although inter‐ and intraobserver reliability were not assessed in the present study, previous studies have reported good to excellent reliability for the Sugaya classification when used in similar clinical contexts.^34^

### Statistical Analysis

The sample size was first estimated for a 2‐sample comparison of means assuming a clinically relevant difference of 10 points in the Constant score at 1 year,^36^ a standard deviation of 16 points,^8^ 2‐sided *α* = 0.05, 80% power, and a 1:3 allocation ratio. This yielded 27 cases and 81 controls (108 patients total) under an independence assumption. Because the final analysis uses a mixed‐effects linear model with 1:3 matched clusters (1 case + ~3 controls per cluster), we inflated the required sample size using a design effect of 1+(m−1)ρ, with *m* = 4 and an assumed intracluster correlation *ρ* = .05, giving a factor of 1.15. The resulting target sample size was therefore approximately 32 cases in the anterior tear group and 96 controls in the posterosuperior tear group (128 patients total).

Descriptive statistics were first performed to summarize patient demographics, lesion characteristics, and surgical variables. Continuous variables were expressed as means with standard deviations and categorical variables as counts with percentages. Between‐group comparisons for continuous variables were performed using unpaired Student's *t* tests (Welch correction applied when variances were unequal) and using *χ* ^2^ or Fisher's exact tests, as appropriate, for categorical variables. The proportions of patients achieving or exceeding the minimal clinically important difference were calculated for each patient‐reported outcome measure using the following published anchor‐based thresholds: Constant score, 10 points;^36^ ASES score, 21 points; pain visual analog scale, 1.5 points; and SANE score, 12 points.^37^

To reduce potential confounding, we performed propensity score matching based on variables selected according to both clinical expertise and statistical relevance, specifically variables that differed between the 2 groups and that could plausibly influence the 1‐year Constant score outcome: age, gender, traumatic origin, tendon retraction, preoperative IR, and Constant scores. We made particular effort to exclude potential mediators or collider variables from the matching process to avoid adjusting on factors lying on the causal pathway between group assignment and outcome (e.g., surgical characteristics). Matching was performed in a 1:3 ratio using nearest‐neighbor matching without replacement (caliper 0.2).

After matching, covariate balance was assessed using standardized mean differences. In the descriptive tables, unweighted standardized mean differences below 0.2 were considered indicative of adequate balance, whereas the Love plot displays weighted standardized mean differences, all below 0.1, confirming optimal covariate balance after matching. Postmatching analyses were performed using a linear mixed‐effects model with the matching cluster included as a random effect to account for within‐pair correlation. The fixed effect of interest was the group variable, with results expressed as estimated coefficients and 95% confidence intervals. Although propensity score matching aimed to balance baseline characteristics between groups, a slight residual imbalance remained for smoking and diabetes, 2 clinically relevant factors known to influence tendon healing and postoperative functional outcomes. Both variables were therefore considered for inclusion in the mixed‐effects model to control for potential residual confounding. However, because smoking and diabetes were highly collinear in the matched cohort (all diabetic patients were smokers), only smoking status was retained in the model to avoid multicollinearity and unstable estimates. Model assumptions were verified, including assessment of residual normality, homoscedasticity, and multicollinearity (using variance inflation factors), to ensure the validity of the model estimates.

All statistical analyses were 2‐tailed with an *α* level set at 0.05. Analyses were conducted using R software (R Foundation for Statistical Computing, Vienna, Austria), version 4.5.0 (2025‐04‐11).

## RESULTS

A total of 1045 patients were initially screened, of whom 639 met the inclusion criteria and were eligible for matching (569 in the posterosuperior tear group and 70 in the anterior tear group) (Figure 1). After 1:3 propensity score matching, 146 patients with posterosuperior tears were matched to 53 patients with anterior tears. Overall, 44 (80%) exposed patients had a complete 1:3 match, whereas 9 (16%) had 1 or 2 matched controls, reflecting good overall matching efficiency. In the posterosuperior tears group, there were 90 isolated supraspinatus tears (62%), 53 type D tears (36%), and 3 type E tears (2%). Baseline patient demographics and tear characteristics were well balanced between the 2 groups (Table 1, Supporting Information).

**FIGURE 1:**
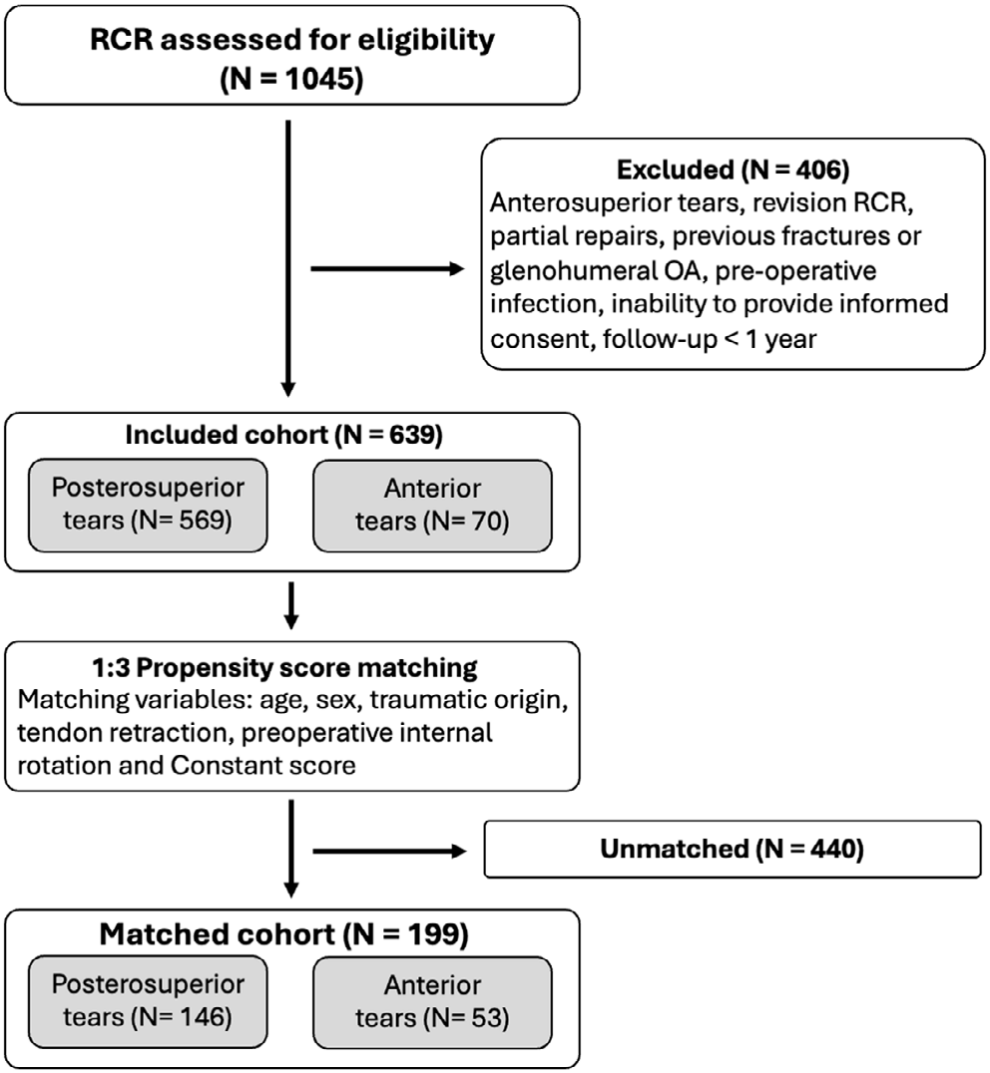
Flowchart illustrating numbers of RCRs assessed for eligibility, reasons for exclusion, factors used for propensity matching, and final included matched cohort numbers. (OA, osteoarthritis; RCR, rotator cuff repair.)

**TABLE 1:** Patient Preoperative Characteristics Between PSG and AG Before and After PSM

	**Before PSM**			**After PSM**		
	**PSG (n = 569)**	**AG (n = 70)**	**SMD**	**PSG (n = 146)**	**AG (n = 53)**	**SMD**
Age, mean (SD)*	54 (10)	52 (9)	0.24	51 (10)	51 (10)	0.01
Male gender, N (%)*	351 (62%)	56 (80%)	0.41	120 (82%)	43 (81%)	0.03
High blood pressure, N/Respond. (%)	67/481 (14%)	8/60 (13%)	0.02	14 (10%)	7 (13%)	0.11
Hypercholesterolemia, N/Respond. (%)	52/481 (11%)	7/60 (12%)	0.03	15 (10%)	7 (13%)	0.09
Diabetes, N/Respond. (%)	97/486 (20%)	14/60 (23%)	0.08	23 (16%)	12 (23%)	0.18
Smokers, N/Respond. (%)	122/486 (25%)	19/60 (32%)	0.15	32 (22%)	16 (30%)	0.19
Workers’ Compensation, N/Respond. (%)	151/567 (27%)	28/69 (41%)	0.30	41 (28%)	19 (36%)	0.17
Traumatic origin, N (%)*	276 (49%)	43 (61%)	0.26	90 (62%)	30 (57%)	0.10
Dominant arm, N (%)	383 (67%)	45 (64%)	0.06	95 (65%)	33 (62%)	0.06
Tendon retraction, N (%)*						
1	346 (61%)	39 (56%)	0.10	107 (73%)	35 (66%)	0.16
2	179 (31%)	8 (11%)	0.50	12 (8%)	4 (8%)	0.02
3	44 (8%)	23 (33%)	0.66	27 (18%)	14 (26%)	0.19
ROM, mean (SD)						
Forward flexion, °	138 (41)	142 (36)	0.10	135 (43)	143 (35)	0.19
ER, °	41 (20)	43 (22)	0.11	40 (20)	42 (21)	0.06
IR (score)*	8 (4)	6 (4)	0.44	6 (4)	6 (4)	0.13
PROM, mean (SD)						
Pain on VAS	56 (18)	57 (18)	0.04	55 (18)	58 (18)	0.18
ASES score	49 (18)	50 (20)	0.06	51 (19)	50 (20)	0.04
Constant score*	49 (19)	50 (21)	0.07	51 (19)	50 (21)	0.04
SANE score	46 (21)	45 (20)	0.10	47 (21)	45 (21)	0.08

AG, anterior tear group; ASES, American Shoulder and Elbow Surgeons; ER, external rotation; IR, internal rotation; N, number of cases; PROM, patient‐reported outcome measure; PSG, posterosuperior tear group; PSM, propensity score matching; ROM, range of motion; SANE, Single Assessment Numeric Evaluation; SD, standard deviation; SMD, standardized mean difference; VAS, visual analog scale; Respond., respondents (in case of missing data for some cases).

* Variable included in the PSM.

As expected, the 2 groups showed significant differences in several surgical parameters (Table 2). The anterior tear group more frequently underwent biceps tenodesis (81% vs 57%, *P *= .002) and sling wearing (92% vs 51%, *P* < .001), whereas the posterosuperior tear group more often had acromioplasty (87% vs 21%, *P* < .001) and double‐row suture techniques (45% vs 4%, *P* < .001). Clavicle resection rates were comparable between groups (13% vs 9%, *P *= .661).

**TABLE 2:** Perioperative Characteristics Between PSG and AG

	**PSG (n = 146)**	**AG (n = 53)**	***P*** **Value**
Clavicle resection, N (%)	19 (13%)	5 (9%)	.661
LHB procedure, N (%)			.002
No touch	22 (15%)	1 (2%)	
Tenodesis	83 (57%)	43 (81%)	
Tenotomy	41 (28%)	9 (17%)	
Acromioplasty, N (%)	127 (87%)	11 (21%)	<.001
Suture technique, N (%)			<.001
Single row	80 (55%)	51 (96%)	
Double row	66 (45%)	2 (4%)	
Sling wearing, N (%)	74 (51%)	49 (92%)	<.001

AG, anterior tear group; LHB, long head of the biceps; PSG, posterosuperior tear group.

As shown in Table 3, the distribution of Sugaya classification grades at 6 months did not differ statistically between the anterior and posterosuperior groups. Similarly, no statistically significant between‐group differences were observed for any range‐of‐motion parameter or patient‐reported outcome measure at 1 year postoperatively. The proportions of patients achieving or exceeding the minimal clinically important difference were also comparable between the posterosuperior and anterior groups for the Constant score (76% vs 81%, *P* = .569), ASES score (69% vs 66%, *P* = .823), pain visual analog scale (92% vs 96%, *P* = .525), and SANE score (77% vs 85%, *P* = .291). In the adjusted mixed‐effects model, the tear group (posterosuperior vs anterior) was not significantly associated with the postoperative Constant score (*β* = 1.7, 95% CI −3.0 to 6.3, *P* = .485).

**TABLE 3:** Patient Outcomes Between PSG and AG

	**PSG (n = 146)**	**AG (n = 53)**	***P*** **Value**
ROM, mean (SD)			
Forward flexion, °	154 (21)	157 (20)	.298
ER, °	42 (21)	48 (28)	.232
IR (score)	8 (3)	7 (4)	.249
PROM, mean (SD)			
Pain on VAS	18 (18)	18 (18)	.690
ASES score	81 (15)	80 (15)	.626
Constant score	77 (15)	78 (16)	.571
SANE score	80 (15)	81 (16)	.667
Sugaya classification grades, N (%)			.214
1	109 (75)	47 (89)	
2	23 (16)	5 (9)	
3	5 (3)	1 (2)	
4	7 (5)	0 (0)	
5	0 (0)	0 (0)	
Unknown	2 (1)	0 (0)	

AG, anterior tear group; ASES, American Shoulder and Elbow Surgeons; ER, external rotation; IR, internal rotation; N, number of cases; PROM, patient‐reported outcome measure; PSG, posterosuperior tear group; ROM, range of motion; SANE, Single Assessment Numeric Evaluation; SD, standard deviation; VAS, visual analog scale.

## DISCUSSION

In this propensity score‐matched cohort of 199 patients, no difference was observed in the 1‐year postoperative Constant score between posterosuperior and anterior RCRs, thus refuting our initial hypothesis. Secondary outcomes, including ultrasound‐assessed healing at 6 months, as well as ASES, pain, and SANE scores and range of motion (forward flexion, external rotation, and IR) at 1 year, were also comparable between groups, although these analyses may be underpowered and should be interpreted cautiously.

### Demographic Factors

The factors that influence the outcome of RCR surgery are complex and involve numerous confounding variables. Consistent with previously published studies (Table 1), patients with anterior rotator cuff tears tended to be younger, predominantly men,^8^ more often traumatic in nature,^38^ and claimants of Workers’ Compensation before matching.^39^

### Anatomical Factors

There are no anatomical factors that appear to support better healing of anterior RCRs. The footprint for the superior tendinous portion of subscapularis's humeral insertion on the lesser tuberosity is narrow (medial‐to‐lateral distance) for such an important muscle, particularly in the distal portion.^40^ This results in a limited surface of contact between the bone bed and the tendon, allowing for a less significant in growth potential compared with the wider anteroposterior and medial‐to‐lateral length of the posterosuperior rotator cuff footprint.^41^ This could explain the rather high rate of posterosuperior healing found in the current study. On the contrary, Shimokobe et al. reported on a larger series of 102 large and massive rotator cuff tears divided into 3 groups based on tear location.^42^ On statistical testing, they reported that posterosuperior tears had statistically significantly higher rates of radiological failure compared with both the other groups that included anterior cuff.^42^ However, a clear distinction was not made in this study between posterosuperior and anterior RCRs, making direct comparison problematic.

### Biological Factors

Based on Codman's cadaver studies and operative findings, it has been commonly believed that anterior RCRs tend to have a better healing rate than posterosuperior repairs. This belief is rooted in the idea that the hypovascular nature of the superior rotator cuff can lead to microcirculatory disturbances, which may predispose it to tears. Codman's work has been supported by subsequent cadaver perfusion studies that have identified areas of hypovascularity, implicating this as a factor in the development of cuff tears.^43^, ^44^ However, more recent studies, using modern technologies such as laser Doppler flowmetry analysis to assess the blood supply of the rotator cuff, have been unable to show a functional hypoperfusion area or the so‐called “critical zone” in the superior normal cuff when compared with the anterior cuff. Therefore, it does not seem that biological factors, such as vascularization, can explain the discrepancies in healing rates between various locations found in previous studies.^42^

### Intraoperative Factors: Tendon Retraction and Type of Repair

Posterosuperior tears are generally thought to exhibit greater tendon retraction than anterior tears,^10^ a feature believed to negatively affect functional outcomes and healing.^45^ In the present study, however, no difference in tendon retraction according to the Patte classification^33^ was observed. At least 2 factors may explain this finding. First, retraction of the anterior cuff is difficult to assess. The anterior cuff is particularly prone to failure in continuity, a phenomenon reported by Meyer et al.^46^ Thus, even when the rotator cuff appears, radiologically and intraoperatively, to remain attached to the lesser tuberosity, the tendon may in fact correspond to a grade 3 retraction according to Patte (Figures 2 and 3). Second, although the Patte classification was developed to describe retraction of the superior cuff, it has also been used to report anterior and posterior rotator cuff retraction. However, the absolute distance associated with superior rotator cuff retraction is greater than that typically observed for anterior or posterior retraction, making direct comparison hazardous (Figure 4).

**FIGURE 2:**
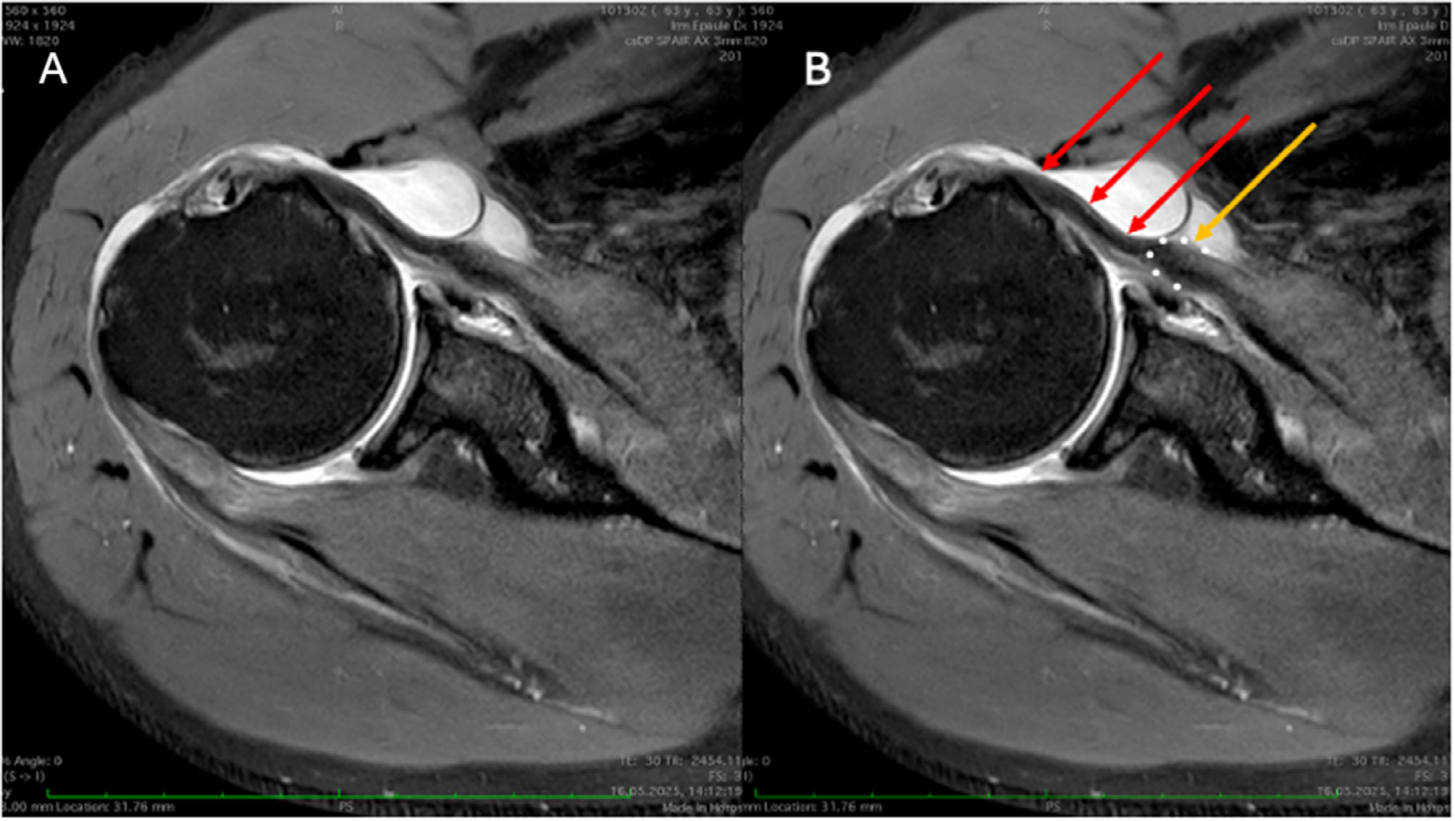
(A) Axial T2‐weighted MRI of a right shoulder. The subscapularis tendon appears to remain attached to the lesser tuberosity. (B) In reality, the tendon stumps (white dots) show a grade 3 retraction. The tissue indicated by the red arrows corresponds to scar tissue bridging the defect, whereas the native tendon is highlighted by the orange arrow. Courtesy of Alexandre Lädermann. (MRI, magnetic resonance imaging.)

**FIGURE 3:**
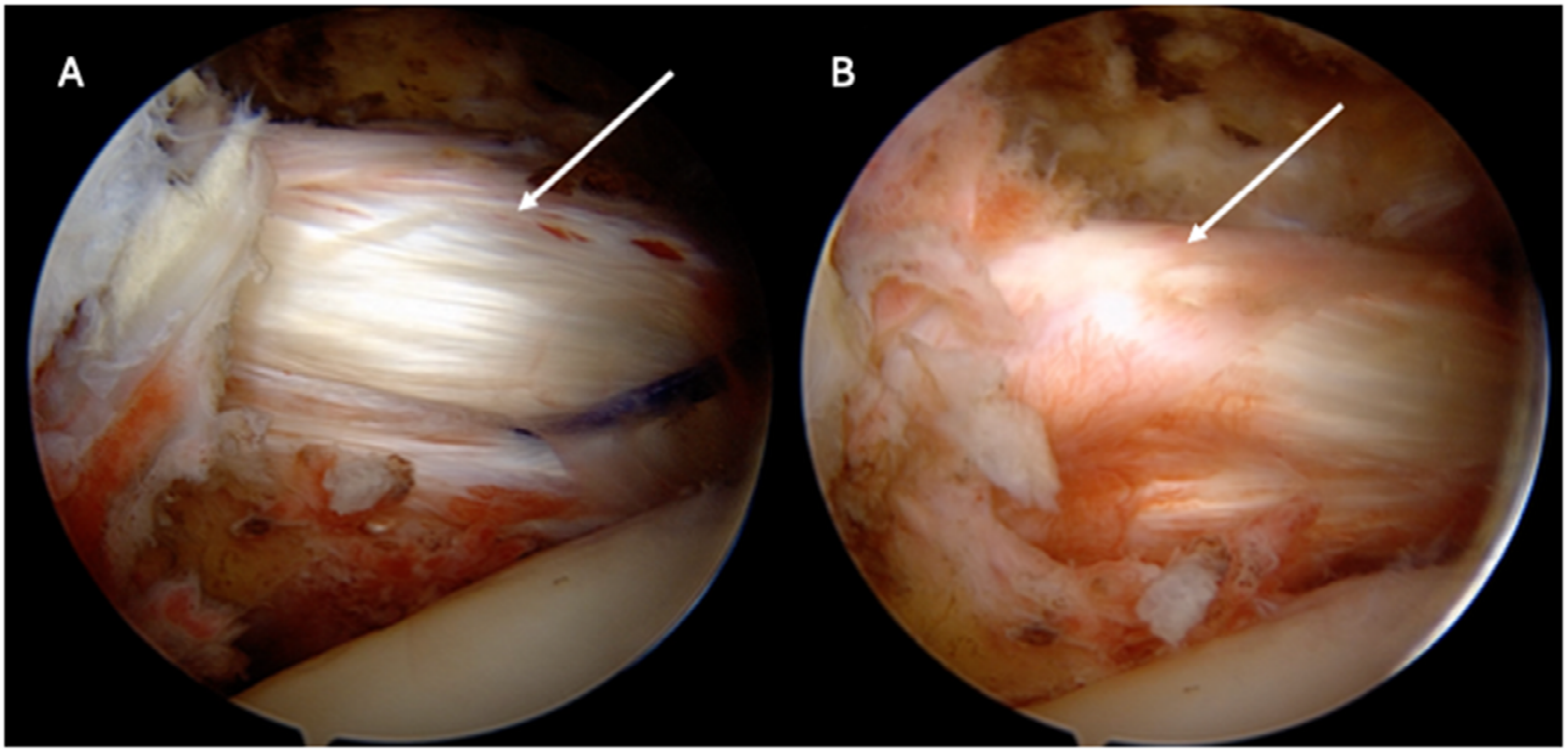
Arthroscopic view of the right shoulder of the same patient (Figures 1 and 3) from a posterior viewing portal. A No. 2 Vicryl traction suture has been passed through the subscapularis tendon. (A) Without traction, the tendon lacks its normal smooth contour and appears disorganized and delaminated. (B) Gentle traction on the suture exposes the lateral portion of the tendon, surrounded by its normal smooth layer. The intact tendon was retracted beyond the anterior glenoid rim. Courtesy of Alexandre Lädermann.

**FIGURE 4:**
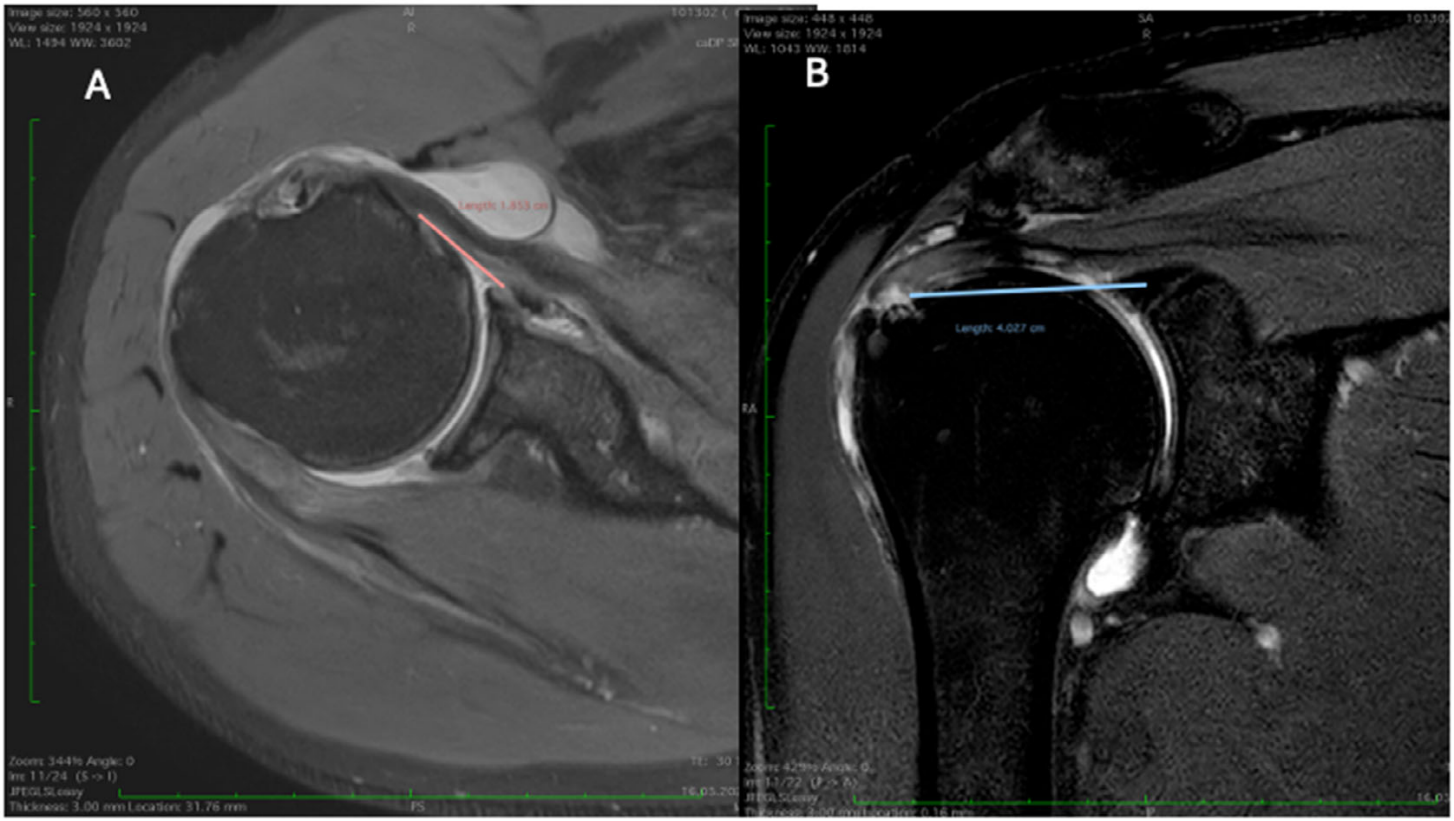
(A) Axial T2‐weighted MRI of a right shoulder. A grade 3 retraction of the subscapularis corresponds to tendon retraction of less than 2 cm from its medial footprint (red line). (B) Coronal T2‐weighted MRI of the same shoulder. A grade 3 retraction of the supraspinatus corresponds to a 4‐cm medial retraction from its medial footprint (blue line). Courtesy of Alexandre Lädermann. (MRI, magnetic resonance imaging.)

In the present study, most RCRs in the posterosuperior group were performed using a double‐row technique, whereas cuff repairs in the anterior group were predominantly performed using a single‐row technique. It remains difficult to determine to what extent this intraoperative difference affects clinical and radiological outcomes. Although the literature generally supports double‐row repair for massive posterosuperior rotator cuff tears,^47^, ^48^ these data do not appear to be directly applicable to anterior RCRs.^49^

The strength of this study lies in the systematic nature of the data collection: every tear had identical baseline and intraoperative variables recorded in a meticulous fashion, providing confidence in the completeness and quality of the dataset. In addition, we analyzed a large, homogeneous cohort with strict propensity score matching, thereby increasing the statistical power of this single‐surgeon series with both pre‐ and postoperative radiological and clinical evaluations. Only shoulders with low‐grade fatty infiltration were included, allowing comparison of tears with a reasonable probability of healing. Finally, the clear separation between purely anterior and posterosuperior RCR is important, and this study avoided pooling different tear patterns, unlike previous publications that combined A‐, C‐, and D‐type lesions.^42^

### Limitations

This study is not without limitations. One limitation of this study is its retrospective design. Concomitant procedures (including biceps tenodesis, acromioplasty, and distal clavicle resection) as well as postoperative sling use differed significantly between the 2 groups. Although these factors may independently influence surgical outcomes, they also reflect inherent differences in the standard management of anterior versus posterosuperior tears. Because the aim of the present study was to compare outcomes between these tear patterns as managed in routine clinical practice, these variables were not controlled for, as they may act as mediators on the causal pathway.^50^ Furthermore, because of the marked male predominance in our cohort (approximately 80% in each group), a sex‐disaggregated analysis as recommended by the Sex and Gender Equity in Research guidelines was not feasible, limiting the generalizability of our findings to female patients.

## CONCLUSIONS

In this propensity score‐matched cohort, anterior and posterosuperior arthroscopic RCRs in patients with limited fatty infiltration yielded comparable 1‐year Constant scores in a real‐world setting.

## SUPPORTING INFORMATION

Additional supporting information can be found online in the Supporting Information section.

## DISCLOSURES

The authors (P.C., A.L.) declare the following financial interests/personal relationships which may be considered as potential competing interests: P.C. receives royalties from and is a consultant and paid speaker for Stryker and Enovis; is the co‐founder of Med4Cast and Follow; and is on the board of SECEC and IBSES. A.L. serves as a paid consultant for Arthrex, Stryker, and Medacta; receives royalties from Stryker and Medacta; and is the co‐founder of FORE, Med4Cast, LastPatient, and BeeMed. The other authors (M.S.R., H.B., A.G., A.A., J.Z.) declare that they have no known competing financial interests or personal relationships that could have appeared to influence the work reported in this paper.

## FUNDING

FORE (Foundation for Research and Teaching in Orthopedics, Sports Medicine, Trauma, and Imaging in the Musculoskeletal System) (2023‐74).

Open access publishing facilitated by Universite de Geneve, as part of the Wiley ‐ Universite de Geneve agreement via the Consortium Of Swiss Academic Libraries.

## Supporting information

Supplementary Material
