# Supplementary material for: Posterosuperior and Anterior Rotator Cuff Repairs Yield Comparable 1‐Year Constant Scores in Routine Clinical Practice: A Propensity Score‐Matched Study From LaTour Group
Source: Arthrosc Sports Med Rehabil. 2026 Oct 9:e70104. Online ahead of print. doi: 10.1002/ars2.70104 (PMC13650963; doi:10.1002/ars2.70104)
Supplement: Supplementary file 1 [file ARS2-9999-e70104-s001.pdf]

Supplementary material 1: Love plot showing covariate balance before and after propensity score matching. All weighted standardized mean differences (SMDs) were < 0.1 after matching, indicating excellent balance between groups.
